# Consent in the time of COVID-19

**DOI:** 10.1136/medethics-2020-106402

**Published:** 2020-06-10

**Authors:** Helen Lynne Turnham, Michael Dunn, Elaine Hill, Guy T Thornburn, Dominic Wilkinson

**Affiliations:** 1 Paediatric Critical Care, Oxford University Hospitals NHS Foundation Trust, Oxford, UK; 2 The Ethox Centre, University of Oxford, Oxford, UK; 3 Cardiothoracic Critical Care and Anaesthesia, Oxford University Hospitals NHS Foundation Trust, Oxford, UK; 4 Consultant Cleft and Plastic Surgeon (Spires Cleft Centre), Oxford University Hospitals NHS Foundation Trust, Oxford, UK; 5 Oxford Uehiro Centre for Practical Ethics, University of Oxford, Oxford, UK

**Keywords:** informed consent, allocation of health care resources, autonomy, decision-making, Distributive justice

## Abstract

The COVID-19 pandemic crisis has necessitated widespread adaptation of revised treatment regimens for both urgent and routine medical problems in patients with and without COVID-19. Some of these alternative treatments maybe second-best. Treatments that are known to be superior might not be appropriate to deliver during a pandemic when consideration must be given to distributive justice and protection of patients and their medical teams as well the importance given to individual benefit and autonomy. What is required of the doctor discussing these alternative, potentially inferior treatments and seeking consent to proceed? Should doctors share information about unavailable but standard treatment alternatives when seeking consent? There are arguments in defence of non-disclosure; information about unavailable treatments may not aid a patient to weigh up options that are available to them. There might be justified concern about distress for patients who are informed that they are receiving second-best therapies. However, we argue that doctors should tailor information according to the needs of the individual patient. For most patients that will include a nuanced discussion about treatments that would be considered in other times but currently unavailable. That will sometimes be a difficult conversation, and require clinicians to be frank about limited resources and necessary rationing. However, transparency and honesty will usually be the best policy.

## Introduction

Since the start of the COVID-19 pandemic, many medical systems have needed to divert routine services in order to support the large number of patients with acute COVID-19 disease. For example, in the National Health Service (NHS) almost all elective surgery has been postponed^1^ and outpatient clinics have been cancelled or conducted on-line treatment regimens for many forms of cancer have changed^2^. This diversion inevitably reduces availability of routine treatments for non-COVID-19-related illness. Even urgent treatments have needed to be modified. Patients with acute surgical emergencies such as appendicitis still present for care, cancers continue to be discovered in patients, and may require urgent management. Health systems are focused on making sure that these urgent needs are met. However, to achieve this goal, many patients are offered treatments that deviate from standard, non-pandemic management.

Deviations from standard management are required for multiple factors such as:

1. Limited resources (staff and equipment reallocated).
2. Risk of nosocomial acquired infection in high-risk patients.
3. Increased risk for medical staff to deliver treatments due to aerosolisation^1^.
4. Treatments requiring intensive care therapy that is in limited availability.
5. Operative procedures that are long and difficult or that are technically challenging if conducted in personal protective equipment. The outcomes from such procedures may be worse than in normal circumstances.
6. Treatments that render patients more susceptible to COVID-19 disease, for example chemotherapy.

There are many instances of compromise, but some examples that we are aware of include open appendectomy rather than laparoscopy to reduce risk of aerosolisation^3^ and offering a percutaneousCoronary intervention (PCI) rather than coronary artery bypass grafting (CABG) for coronary artery disease, to reduce need for intensive care. Surgery for cancers ordinarily operated on urgently maybe deferred for up to 3 months^4^ and surgery might be conducted under local anaesthesia that would typically have merited a general anaesthetic (both to reduce the aerosol risk of General anaesthesia, and because of relative lack of anaesthetists).

The current emergency offers a unique difficulty: a significant number of treatments with proven benefit might be unavailable to patients while those alternatives that are available are not usually considered best practice and might be actually inferior. In usual circumstances, where two treatment options for a particular problem are considered appropriate, the decision of which option to pursue would often depend on the personal preference of the patient.

But during the pandemic what is ethically and legally required of the doctor or medical professional informing patients about treatment and seeking their consent? In particular, do health professionals need to make patients aware of the usual forms of treatment that they are not being offered in the current setting?

We consider two theoretical case examples:

### Case 1

Jenny^2^ is a model in her mid-20s who presents to hospital at the peak of the COVID-19 pandemic with acute appendicitis. Her surgeon, Miss Schmidt, approaches Jenny to obtain consent for an open appendectomy. Miss Schmidt explains the risks of the operative procedure, and the alternative of conservative management (with intravenous antibiotics). Jenny consents to the procedure. However, she develops a postoperative wound infection and an unsightly scar. She does some research and discovers that a laparoscopic procedure would ordinarily have been performed and would have had a lower chance of wound infection. She sues Miss Schmidt and the hospital trust where she was treated.

### Case 2

June^2^s a retired teacher in her early 70s who has well-controlled diabetes and hypertension. She is active and runs a local food bank. Immediately prior to the pandemic lockdown in the UK June had an episode of severe chest pain and investigations revealed that she has had a non-ST elevation myocardial infarction. The cardiothoracic surgical team recommends that June undergo a PCI although normally her pattern of coronary artery disease would be treated by CABG. When the cardiologist explains that surgery would be normally offered in this situation, and is theoretically superior to PCI, June’s husband becomes angry and demands that June is listed for surgery.

### In favour of non-disclosure

It might appear at first glance that doctors should obviously inform Jenny and June about the usual standard of care. After all, consent cannot be informed if crucial information is lacking. However, one reason that this may be called into question is that it is not immediately clear how it benefits a patient to be informed about alternatives that are not actually available? In usual circumstances, doctors are not obliged to inform patients about treatments that are performed overseas but not in the UK. In the UK, for example, there is a rigorous process for assessment of new treatments (not including experimental therapies). Some treatments that are available in other jurisdictions have not been deemed by the National Institute for Health and Care Excellence (NICE) to be sufficiently beneficial and cost-effective to be offered by the NHS. It is hard to imagine that a health professional would be found negligent for not discussing with a patient a treatment that NICE has explicitly rejected. The same might apply for novel therapies that are currently unfunded pending formal evaluation by NICE.

Of course, the difference is that the treatments we are discussing have been proven (or are believed) to be beneficial and would normally be provided. The Montgomery Ruling of 2015 in the UK established that patients must be informed of material risks of treatment and reasonable alternatives to treatment. The *Bayley –v- George Eliot Hospital NHS Trust* 5case established that those reasonable alternative treatments must be ‘appropriate treatment’ not just a ‘possible treatment’^6^. In the current crisis, many previously standard treatments are no longer appropriate given the restrictions outlined. In other circumstances they are appropriate; during a pandemic they are no longer appropriate, even if they become appropriate again at some unknown time in the future.

In both ethical and legal terms, it is widely accepted that, for consent to be valid, if must be given voluntarily by a person who has capacity to consent and who understands the nature and risks of the treatment. A failure to obtain valid consent, or performing interventions in the absence of consent, could result in criminal proceedings for assault. Failing to provide adequate information in the consent process could support a claim of negligence. Ethically, adequate information about treatments is essential for the patient to enable them to weigh up options and decide which treatments they wish to undertake. However, information about unavailable treatments arguably does not help the patient make an informed decision because it does not give them information that is relevant to consenting or to refusal of treatment that is actually available. If Miss Schmidt had given Jenny information about the relative benefits of laparoscopic appendectomy, that could not have helped Jenny’s decision to proceed with surgery. Her available choices were open appendectomy or no surgery. Moreover, as the case of June highlights, providing information about alternatives may lead them to desire or even demand those alternative options. This could cause distress both to the patient and the health professional (who is unable to acquiesce).

Consideration might also be paid to the effect on patients of disclosure. How would it affect a patient with newly diagnosed cancer to tell them that an alternative, perhaps better therapy, might be routinely available in usual circumstances but is not available now?

There is provision in the Montgomery Ruling, in rare circumstances, for therapeutic exception. That is, if information is significantly detrimental to the health of a patient it might be omitted. We could imagine a version of the case where Jenny was so intensely anxious about the proposed surgery that her surgeon comes to a sincere belief that discussion of the laparoscopic alternative would be extremely distressing or might even lead her to refuse surgery. In most cases, though, it would be hard to be sure that the risks of disclosing alternative (non-available) treatments would be so great that non-disclosure would be justified.

### In favour of disclosure

In the UK, professional guidance issued by the GMC (General Medical Council) requires doctors to take a personalised approach to information sharing about treatments by sharing ‘with patients the information they want or need in order to make decisions’. The *Montgomery* judgement of 2015^7^ broadly endorsed the position of the GMC, requiring patients to be told about any *material* risks and reasonable alternatives relevant to the decision at hand. The Supreme Court clarifies that materiality here should be judged by reference to a new two-limbed test founded on the notions of the ‘reasonable person in the patient’s position’ and the ‘particular patient’. One practical test might be for the clinician to ask themselves whether patients in general, or this particular patient might wish to know about alternative forms of treatment that would usually be offered.

The GMC has recently produced pandemic-specific guidance^8^ on consent and decision-making, but this guidance is focused on managing consent in COVID-19-related interventions. While the GMC takes the view that its consent guidelines continue to apply as far as is practical, it also notes that the patient is enabled to consider the ‘reasonable alternatives’, and that the doctor is ‘open and honest with patients about the decision-making process and the criteria for setting priorities in individual cases’.

In some situations, there might be the option of delaying treatment until later; when other surgical procedures are possible. In that setting, it would be important to ensure that the patient is aware of those future options (including the risks of delay). For example, if Jenny had symptomatic gallstones, her surgeons might be offering an open cholecystectomy now or the possibility of a laparoscopic surgery at some later point. Understanding the full options open to her now and in the future may have considerable influence on Jenny’s decision. Likewise, if June is aware that she is not being offered standard treatment she may wish to delay treatment of her atherosclerosis until a later date. Of course, such a delay might lead to greater harm overall. However, it would be ethically permissible to delay treatment if that was the patient’s informed choice (just as it would be permissible for the patient to refuse treatment altogether).

In the appendicitis case, Jenny does not have the option for delaying her treatment, but the choice for June is more complicated, between immediate PCI which is a second-best treatment versus waiting for standard therapy. Immediate surgery also raises a risk of acquiring nosocomial COVID-19 infection and June is in an age group and has comorbidities that put her at risk of severe COVID-19 disease. Waiting for surgery leaves June at risk of sudden death. For an active and otherwise well patient with coronary disease like June, PCI procedure is not as good a treatment as CABG and June might legitimately wish to take her chances and wait for the standard treatment. The decision to operate or wait is a balance of risks that only June is fully able to make. Patients in this scenario will take different approaches. Patients will need different amounts of information to form their decisions, many patients will need as much information as is available including information about procedures not currently available to make up their mind.

June’s husband insists that she should receive the best treatment, and that she should therefore be listed for CABG. Although this treatment would appear to be in June’s best interests, and would respect her autonomy, those ethical considerations are potentially outweighed by distributive justice. The COVID-19 pandemic of 2020 is being characterised by limitations; liberties curtailed and choices restricted, this is justified by a need to protect healthcare systems from demand exceeding availability. While resource allocation is always a relevant ethical concern in publicly funded healthcare systems, it is a dominant concern in a setting where there is a high demand for medical care and scare resources.

It is well established that competent adult patients can consent to or refuse medical treatment but they cannot demand that health professionals provide treatments that are contrary to their professional judgement or (even more importantly) would consume scarce healthcare resources. In June’s case, agreeing to perform CABG at a time when large numbers of patients are critically ill with COVID-19 might mean that another patient is denied access to intensive care (and even dies as a result). Of course, it may be that there are actually available beds in intensive care, and June’s operation would not directly lead to denial of treatment for another patient. However, that does not automatically mean that surgery must proceed. The hospital may have been justified in making a decision to suspend some forms of cardiac surgery. That could be on the basis of the need to use the dedicated space, staff and equipment of the cardiothoracic critical care unit for patients with COVID-19. Even if all that physical space is not currently occupied if may not be feasible or practical to try to simultaneously accommodate some non-COVID-19 patients. (There would be a risk that June would contract COVID-19 postoperatively and end up considerably worse off than she would have been if she had instead received PCI.) Moreover, it seems problematic for individual patients to be able to circumvent policies about allocation of resources purely on the basis that they stand to be disadvantaged by the policy.

Perhaps the most significant benefit of disclosure of non-options is transparency and honesty. We suggest that the main reason why Miss Schmidt ought to have included discussion of the laparoscopic alternative is so that Jenny understands the reasoning behind the decision. If Miss Schmidt had explained to Jenny that in the current circumstances laparoscopic surgery has been stopped, that might have helped her to appreciate that she was being offered the best available management. It might have enabled a frank discussion about the challenges faced by health professionals in the context of the pandemic and the inevitable need for compromise. It may have avoided awkward discussions later after Jenny developed her complication.

Transparent disclosure should not mean that patients can demand treatment. But it might mean that patients could appeal against a particular policy if they feel that it has been reached unfairly, or applied unfairly. For example, if June became aware that some patients were still being offered CABG, she might (or might not) be justified in appealing against the decision not to offer it to her. Obviously such an appeal would only be possible if the patient were aware of the alternatives that they were being denied.

For patients faced by decisions such as that faced by June, balancing risks of either option is highly personal; individuals need to weigh up these decisions for them and require all of the information available to do so. Some information is readily available, for example, the rate of infection for Jenny and the risk of death without treatment for June. But other risks are unknown, such as the risk of acquiring nosocomial infection with COVID-19. Doctors might feel discomfort talking about unquantifiable risks, but we argue that it is important that the patient has all available information to weigh up options for them, including information that is unknown.

## Conclusion

In a pandemic, as in other times, doctors should ensure that they offer appropriate medical treatment, based on the needs of an individual. They should aim to provide available treatment that is beneficial and should not offer treatment that is unavailable or contrary to the patient best interests. It is ethical; indeed it is vital within a public healthcare system, to consider distributive justice in the allocation of treatment. Where treatment is scarce, it may not be possible or appropriate to offer to patients some treatments that would be beneficial and desired by them.

Informed consent needs to be individualised. Doctors are obliged to tailor their information to the needs of an individual. We suggest that in the current climate this should include, for most patients, a nuanced open discussion about alternative treatments that would have been available to them in usual circumstances. That will sometimes be a difficult conversation, and require clinicians to be frank about limited resources and necessary rationing. However, transparency and honesty will usually be the best policy.

## Footnotes

The concern is that medical staff may contract COVID-19 from a patient who has early asymptomatic illness and another coincidental medical problem.

These are hypothetical cases (although a composite of our clinical experience).
